# Structure and function study of the complex that synthesizes *S*-adenosylmethionine

**DOI:** 10.1107/S2052252514012585

**Published:** 2014-06-12

**Authors:** Ben Murray, Svetlana V. Antonyuk, Alberto Marina, Sebastiaan M. Van Liempd, Shelly C. Lu, Jose M. Mato, S. Samar Hasnain, Adriana L. Rojas

**Affiliations:** a Molecular Biophysics Group, Institute of Integrative Biology, Faculty of Health and Life Sciences, University of Liverpool, L69 7ZX, England; b Structural Biology Unit CIC bioGUNE, Parque Tecnológico de Bizkaia, 48160 Derio, Bizkaia, Spain; c Metabolomics Unit, CIC bioGUNE, CIBERehd, Parque Tecnológico de Bizkaia, 48160 Derio, Bizkaia, Spain; d Division of Gastroenterology and Liver Diseases, USC Research Center for Liver Diseases, USC–UCLA Research Center for ALPD and Cirrhosis, Keck School of Medicine, Los Angeles, California, CA 90033, USA

**Keywords:** methionine adenosyltransferases, cell growth, liver cancer, X-ray scattering, methylation, drug design

## Abstract

*S*-Adenosylmethionine (SAMe) is the principal methyl donor of the cell and is synthesized *via* an ATP-driven process by methionine adenosyltransferase (MAT) enzymes. It is tightly linked with cell proliferation in liver and colon cancer. In humans, there are three genes, *mat1A, mat2A* and *mat2B*, which encode MAT enzymes. *mat2A* and *mat2B* transcribe MATα2 and MATβ enzyme subunits, respectively, with catalytic and regulatory roles. The MATα2β complex is expressed in nearly all tissues and is thought to be essential in providing the necessary SAMe flux for methylation of DNA and various proteins including histones. In human hepatocellular carcinoma *mat2A* and *mat2B* genes are upregulated, highlighting the importance of the MATα2β complex in liver disease. The individual subunits have been structurally characterized but the nature of the complex has remained elusive despite its existence having been postulated for more than 20 years and the observation that MATβ is often co-localized with MATα2. Though SAMe can be produced by MAT(α2)_4_ alone, this paper shows that the *V*_max_ of the MATα2β complex is three- to fourfold higher depending on the variants of MATβ that participate in complex formation. Using X-ray crystallography and solution X-ray scattering, the first structures are provided of this 258 kDa functional complex both in crystals and solution with an unexpected stoichiometry of 4α2 and 2βV2 subunits. It is demonstrated that the N-terminal regulates the activity of the complex and it is shown that complex formation takes place surprisingly *via* the C-terminal of MATβV2 that buries itself in a tunnel created at the interface of the MAT(α2)_2_. The structural data suggest a unique mechanism of regulation and provide a gateway for structure-based drug design in anticancer therapies.

## Abstract 2

MAT2 complex is expressed in nearly all tissues and is essential in providing the necessary SAMe flux for methylation of DNA and various proteins including histones. X-ray crystallography and solution X-ray scattering structures of this complex show an unexpected stoichiometry, offering a unique mechanism of regulation and thus providing a gateway for structure-based drug design in anticancer therapies including liver disease.

## 1. Introduction   

Transmethylation, the transfer of a methyl group between molecules, plays a central role in fundamental biological processes such as cell growth, gene expression and apoptosis (Lu & Mato, 2012 ▶). The *S*-adenosylmethionine (SAMe) molecule (Landgraf & Booker, 2013 ▶), which is synthesized by methionine adenosyltransferase (MAT), is the main source of methyl groups in all living organisms. MAT enzymes are conserved from bacteria to mammals, thus highlighting their essential regulatory function in maintaining the appropriate levels of SAMe. 

**Figure 1:**
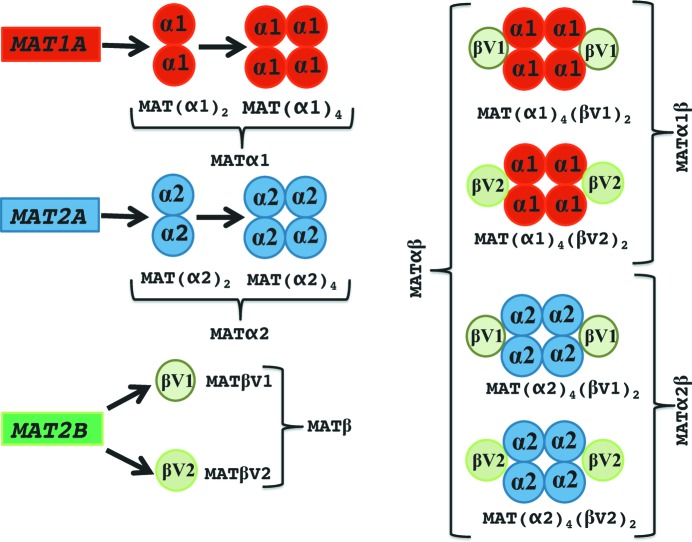
Schematic representation of the oligomeric states of mammalian MAT enzymes. The mammalian genes *mat1A* and *mat2A* produce the catalytic subunits MATα1 and MATα2, respectively, sharing 84% sequence homology. The MATα1 and MATα2 subunits can be found organized as dimers and tetramers. The MATα1 dimer and tetramer are known as MATIII and MATI, respectively. On the other hand, *mat2B* encodes the regulatory subunit for which there are two major isoforms, MATβV1 and MATβV2. The MAT(α2)_4_(βV2)_2_ complex consists of a MATα2 tetramer flanked by two MATβV2 subunits. In this study we were able to assemble *in vitro* three different MATαβ complexes: MAT(α2)_4_(βV1)_2_, MAT(α2)_4_(βV2)_2_ and MAT(α1)_4_(βV1)_2_ (the last two complexes are described for the first time).

Mammals express three MAT genes, *mat1A*, *mat2A* and *mat2B*; the former two encode two homologous MAT catalytic subunits, MATα1 and MATα2, and the last one encodes the regulatory subunit MATβ. Association of MATα and MATβ subunits results in the formation of the MATαβ complex. Recently, it was shown that there exist two major splicing variants of the *mat2B* gene that encode two proteins MATβV1 and MATβV2. Both isoforms interact with MATα2 (Yang *et al.*, 2008 ▶; Xia *et al.*, 2010 ▶), revealing the existence of two new complexes: MATα2βV1 and MATα2βV2 (Fig. 1 ▶). MATα2 expression confers a cell growth advantage and is considered increasingly important for differentiation and apoptosis (Lu & Mato, 2012 ▶), for instance in human hepatocellular carcinoma (HCC) (Yang *et al.*, 2008 ▶), colon cancer (Chen *et al.*, 2007 ▶) and leukaemic cells (Attia *et al.*, 2008 ▶). MATα2β interacts with a large variety of proteins both in the nucleus and cytoplasm of mammalian cells, including HuR (Xia *et al.*, 2010 ▶), an mRNA-binding protein known to stabilize the mRNA of cyclins; GIT1 (Peng *et al.*, 2013 ▶), a scaffold protein that activates ERK1/2; MafK (Katoh *et al.*, 2011 ▶), a Maf family transcription factor; and many others. Global histone methylation decreases upon MAT knockdown in *Caenorhabditis elegans* causing the release of heterochromatin from the nuclear periphery (Towbin *et al.*, 2012 ▶). Recently it has been demonstrated that threonine and SAMe metabolism are coupled in pluripotent stem cells, resulting in regulation of histone methylation (Shyh-Chang *et al.*, 2013 ▶). Similarly, disruption in mice of GNMT, the enzyme that catalyses the methylation of glycine to synthesize sarcosine, and overexpression in ovary cancer cells of NNMT, the enzyme that synthesizes 1-methylnico­tin­amide by methylation of nicotinamide, cause aberrant DNA, histone and phospholipid methylation by altering cellular SAMe (Martínez-Chantar *et al.*, 2008 ▶; Ulanovskaya *et al.*, 2013 ▶). GNMT knockdown has been shown to attenuate prostate cancer (Sreekumar *et al.*, 2009 ▶). This has led to an emerging paradigm in enzyme regulation, where MATα2β synthesizes SAMe locally to support specific methylation reactions (Kaelin & McKnight, 2013 ▶; Gibson & Kraus, 2011 ▶; Martinez-Una *et al.*, 2013 ▶).

The synthesis of SAMe has been proposed to follow an SN_2_ catalytic mechanism (Markham *et al.*, 1987 ▶) that has gained structural support from the crystallographic analyses of MAT catalytic subunits bound to various ligands (Komoto *et al.*, 2004 ▶; González *et al.*, 2003 ▶; Shafqat *et al.*, 2013 ▶). Briefly, the reaction is initiated by the sulfur atom of the methionine which carries a nucleophilic attack against the C5′ atom of ATP followed by the hydrolysis of the tripolyphosphate (PPPi) (Supplementary Fig. S1A). Despite the central role of MATα2β, the nature of the complex has remained elusive and its structural elucidation a challenge. Here we report the crystallographic and solution X-ray scattering structures as well as biophysical analysis of the MATα2β complexes, providing the basis for fresh insight into this widely utilized system in biology.

## 2. Results and discussion   

### 2.1. MATβ isoforms interact with MAT(α2)_2_ through the C-terminal region   

**Figure 2:**
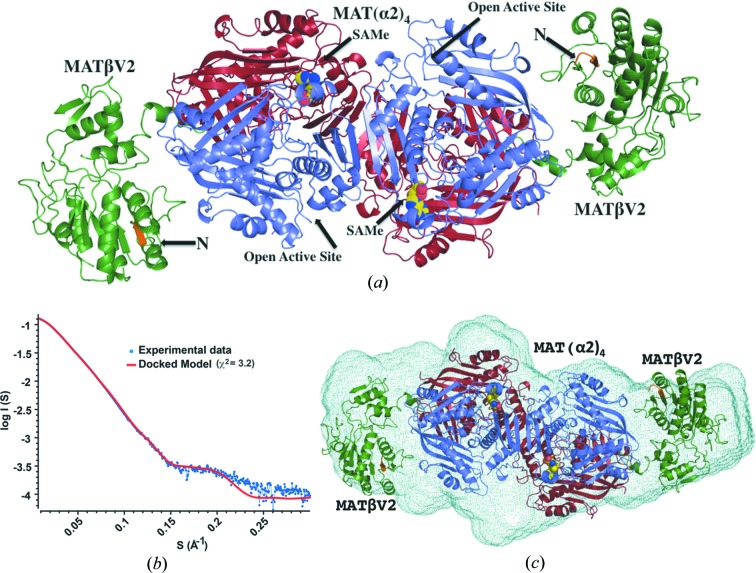
Structure of the MAT(α2)_4_(βV2)_2_ complex. (*a*) Crystallographic structure of the MAT(α2)_4_(βV2)_2_ complex. Two MATα2 monomers with visible gating loops are coloured in slate whereas the other two MATα2 monomers are coloured in red. MATβV2 is coloured in green with the first visible residues at its N-terminal in orange. The reaction product (SAMe) is represented by spheres. (*b*) SAXS of the MAT(α2)_4_(βV2)_2_ complex, the experimental spectrum (blue) is shown with a simulated fit (red) obtained from the crystal structure (*q*_max_ = 0.3 Å^−1^) (χ^2^ = 3.2). The radius of gyration, *R*_g_ = 50.1 ± 0.05 Å, was estimated from the low-angle scattering region by a Guinier plot. The distance distribution function, *P*(*r*), with maximum linear dimension *D*_max_ = 187 Å. We interpret the higher χ^2^ value as arising in part from the flexibility of the interaction and the missing residues at the N-terminus of both MATβ subunits in the crystal structure. This is supported when SAXS data of the MATα2βV1Δ16 mutant (slightly better quality data, data not shown) are used with the model from the crystal structure (χ^2^ = 1.7). (*c*) The *ab initio* shape reconstruction of MAT(α2)_4_(βV2)_2_ by *DAMMIN* using *P*1 symmetry, showing a good agreement between the predicted molecular shape (light green mesh) and the crystal structure (*R*_g_ = 50.7) (cartoon), after alignment by *SUPCOMB* (Kozin & Svergun, 2001 ▶) (NSD = 0.93).

Crystallographic structures of the 258 kDa MATα2βV2 complex determined in different forms to a resolution ranging from 2.35 to 3.3 Å reveal that the complex consists of a 185 kDa MATα2 tetramer which is flanked by two MATβV2 subunits of 36.5 kDa each (Fig. 2 ▶ *a*). The complex has four active sites located at the interface of the MATα2 dimers. The oligomeric state of our crystallographic structure is different to the suggested tetrameric form [MAT(α2)_2_(β)_2_] (Kotb & Kredich, 1985 ▶) or the recently proposed model in which MATαβ was assumed to be a trimer [MAT(α2)_2_(β)_1_] (González *et al.*, 2012 ▶).

In the first study Kotb and Kredich used analytical ultracentrifugation analysis to estimate the molecular weight of the complex whereas in the second study Gonzales *et al.* used ITC (isothermal titration calorimetry) to investigate its stoichiometry which matches our model in a 2:1 ratio of MATα2:MATβ subunits. In order to confirm that the crystallographic oligomer is representative of the solution state and is thus physiologically relevant, we performed small-angle X-ray scattering (SAXS) experiments using the HPLC-integrated SAXS set-up at the SOLEIL synchrotron. The scattering curve of the complex in solution obtained by SAXS is in agreement with the theoretical curve calculated from our 2.35 Å crystal structure (χ^2^ = 3.2) (Fig. 2 ▶ *b*), confirming that the MATα2β complex in solution does indeed have the same composition and overall conformation as observed in our crystal structures, *i.e.* MAT(α2)_4_(βV2)_2_ (Fig. 2 ▶ *c*).

**Figure 3:**
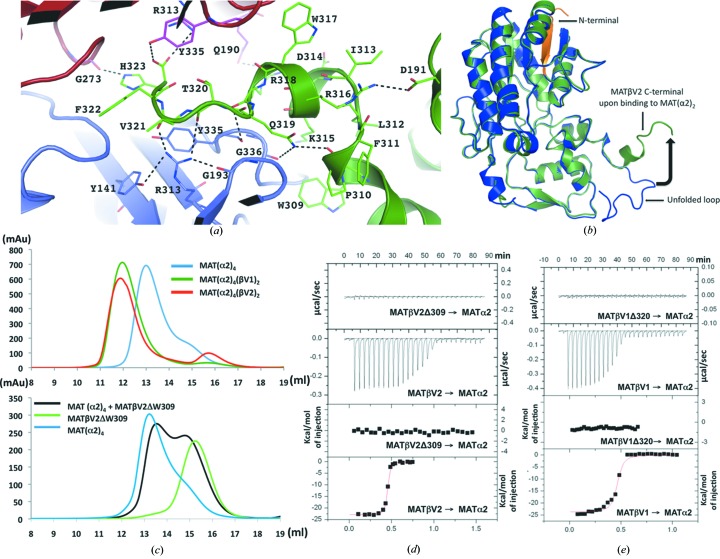
MATβV2 C-terminal interactions with MATα2. (*a*) Each MATβV2 subunit interacts with the core by inserting its C-terminus (green) in a tunnel created at the interface between two MATα2 subunits (slate, red). The residues involved in the interaction are in stick representation (hydrogen bonds in black dotted lines). (*b*) Superposition of MATβ (PDB entry 2ydy) in blue with MATβV2 from the complex with MATα2 (green). The black arrow indicates the conformational change of the unfolded C-terminal loop. (*c*) Gel-filtration profiles for MAT(α2)_4_(βV2)_2_ (red), MAT(α2)_4_(βV1)_2_ (dark green) and MAT(α2)_4_ (blue). For complex formation MATα2 was incubated with both MATβ variants prior to being loaded onto a Superdex 200 10/300 column. The gel-filtration profiles clearly show the shift of the peak that contains the complex (top) and absence of complex formation when MATβV2ΔW309 (black) is used; MAT(α2)_4_ (blue) and MATβV2ΔW309 (light green) were loaded as controls (bottom). (*d*) ITC of MATα2 with MATβV2; the top graphs represent the differential heat released during the titration of MATβV2ΔW309 or MATβV2 with MATα2. The bottom graphs represent the fitted binding isotherms. (*e*) As in (*d*) ITC of MATα2 with MATβV1ΔW320 or MATβV1.

The crystallographic structure of the MAT(α2)_4_(βV2)_2_ complex reveals that MATβV2 interacts with MATα2 through the insertion of the C-terminal tail of the β subunit into a cavity created at the interface of the MATα2 dimer. Specifically, residues K315 to H323 of MATβV2 establish extensive hydrophobic and polar interactions with side chains of both MATα2 monomers (Fig. 3 ▶ *a*). In the complex, the usually disordered tail of the MATβV2 C-terminal folds into a helical structure (Fig. 3 ▶ *b*), and within the MAT(α2)_2_ binding cavity the interaction generates a dilation of the cleft without any change in the orientation of the side chains consistent with a ‘lock-and-key’ mechanism. In order to establish that the C-terminal tail is indeed the key region of the interaction, we generated a truncated version lacking the last 15 residues at the C-terminus in both variants of MATβ, MATβV1 and MATβV2. These deletions, though preserving the secondary structure, as confirmed by circular dichroism (CD) spectra (Fig. S1B), precluded the assembly of the complex in solution (gel filtration, Fig. 3 ▶ *c*; and ITC, Figs. 3 ▶ *d*, 3 ▶ *e* and Table 1 ▶). Taken together, these data show that MATβV2 and MATβV1 interact with MAT(α2)_2_ through the insertion of the C-terminal tail of the β subunit.

**Figure 4:**
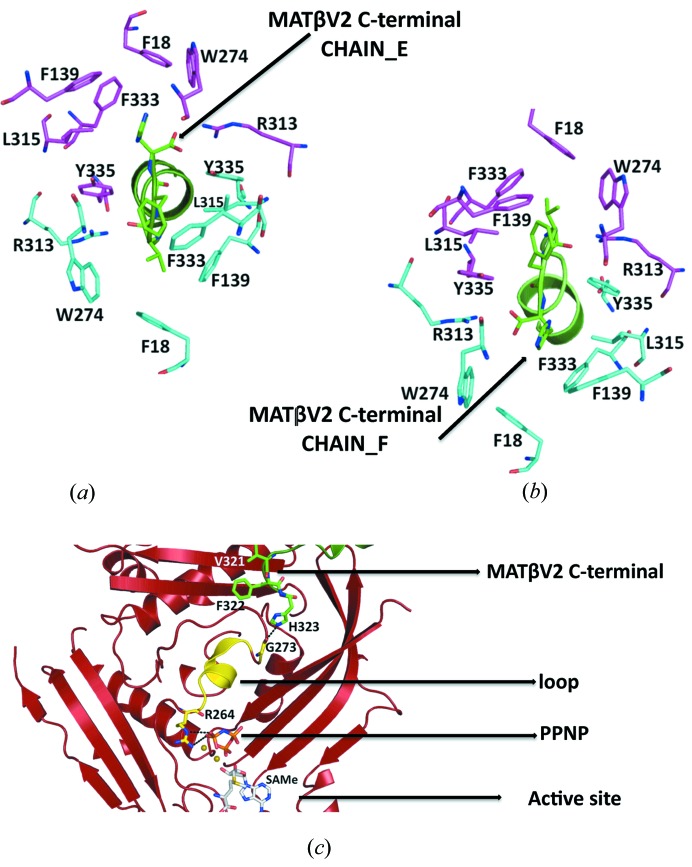
Close view of the tunnel created at the interface of the MATα2 dimer. (*a*) Stick representation of the MATα2 residues at the dimer interface, side chains involved in the interaction with MATβV2 (chain_E in green) are coloured in cyan, the symmetry residues are shown in magenta. (*b*) Side chains involved in the interaction with MATβV2 (chain_F in green) are coloured in magenta, the symmetry residues are shown in cyan. Note that between the conformation represented in (*a*) and (*b*) there is a twofold symmetry. (*c*) Cartoon representation of the MATα2 monomer; the loop that connects the active site with the buried tail of MATβV2 is highlighted in yellow.

Interestingly, the tunnel created at the interface of the MATα2 dimer has a symmetry that allows two possible conformations of the C-terminal MATβV2 (Figs. 4 ▶ *a* and 4 ▶ *b*). The interchangeability of these two orientations could allow certain rotational flexibility of the MATβV2 subunit.

### 2.2. NADP-binding site   

**Figure 5:**
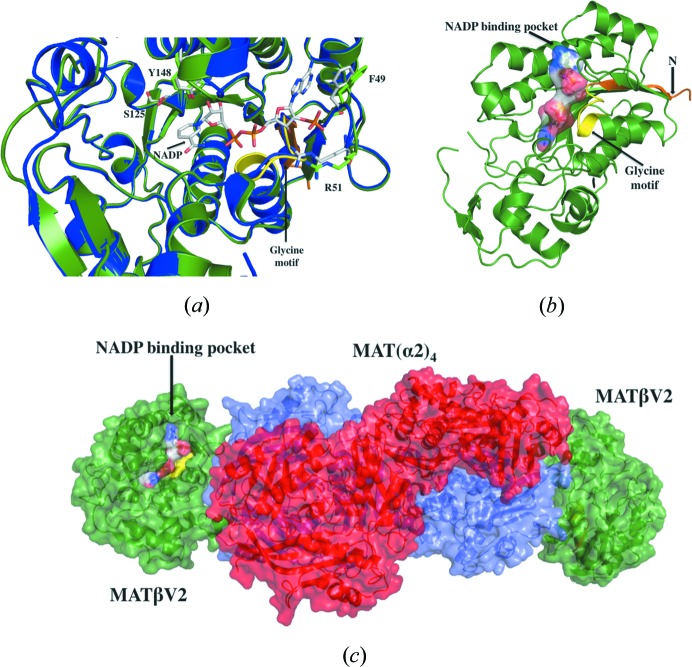
NADP-binding site. (*a*) Superposition of the NADP-bound MATβ structure (PDB entry 2ydx, Shafqat *et al.*, 2013 ▶, in blue) over MATβV2 as it is in the MAT(α2)_4_(βV2)_2_ complex (in green). Residues directly involved in the interaction with NADP are labelled and shown in stick representation. (*b*) Surface representation of the model of an NADP molecule within the binding pocket of MATβV2 from the MAT(α2)_4_(βV2)_2_ complex. (*c*) MAT(α2)_4_ (in red and slate) does not block the access of NADP to MATβV2 (in green).

Previously, it has been shown that NADP binds to a conserved glycine-rich G*XX*G*XX*G motif (G_24_ATG_27_LLG_30_) at the N-terminal domain of MATβ (Shafqat *et al.*, 2013 ▶) (Figs. S1C and 5 ▶ *a*). The mutants lacking residues involved in NADP binding of the β subunit have been shown to be able to form a complex with MATα2 supporting the idea that NADP is not needed to form the complex (González *et al.*, 2012 ▶). In our case, co-crystallization and soaking experiments with an excess of NADP yielded crystals of the MAT(α2)_4_(βV2)_2_ complex without NADP. This observation prompted us to evaluate whether NADP could interact in solution with the preformed complex. ITC experiments confirmed that, though NADP binds to both MATβ isoforms, no interaction could be observed with the MAT(α2)_4_(βV2)_2_ complex (Figs. S2A and S2B; Table 2 ▶). The presence of NADP had no effect on MAT(α2)_4_(βV2)_2_ complex formation as observed by gel filtration and native PAGE experiments (Figs. S2C and S2D). The overall structure of MATβV2 within the MAT(α2)_4_(βV2)_2_ complex has no significant structural changes as compared with the NADP-bound MATβ structure (r.m.s.d. = 0.42 Å) (Figs. 5 ▶ *a* and 5 ▶ *b*). These data taken together suggest that after complex formation the NADP is displaced from its binding pocket. It is possible that NADP binding to MATβV2 is relevant for other functions, such as the interaction with HuR, GIT1, MEK, ERK or MafK (Xia *et al.*, 2010 ▶; Peng *et al.*, 2013 ▶; Katoh *et al.*, 2011 ▶). Furthermore, the observation that MAT(α2)_4_ does not block the binding pocket (Fig. 5 ▶ *c*) suggests that the NADP could bind to the MAT(α2)_4_(βV2)_2_ complex in the context of the interaction with other proteins. In fact, there is evidence suggesting that MAT enzymes are involved in the regulation of many pathways, some of which are chromatin based and some may be independent of SAMe.

### 2.3. Interaction of MATβ isoforms with MATα1   

Looking at the structure of the MAT(α2)_4_(βV2)_2_ complex it is difficult to understand why, *in vivo*, MATβ has so far been found to only interact with MATα2, even though MATα1 and MATα2 have a homology of 84% and share the same folding (Fig. S3). A plausible explanation may be that *in vivo* when the expression of *mat1A* is switched on, *mat2A* and *mat2B* expression is switched off. Thus, whereas in adult hepatocytes *mat1A* is highly expressed and the expression of *mat2A* and *mat2B* is low, in HCC where the expression of *mat2A* and *mat2B* is turned on, as well as the expression of other proteins that interact with MATβ such as HuR, GIT1, MEK, ERK or MafK (Xia *et al.*, 2010 ▶; Peng *et al.*, 2013 ▶; Katoh *et al.*, 2011 ▶), *mat1A* expression is low or absent (Lu & Mato, 2012 ▶). Accordingly, we observed by gel filtration and ITC the formation of the MAT(α1)_4_(βV1)_2_ complex upon incubation of MATβV1 with MATα1 (Figs. S4A and S4B; Table 1 ▶). Remarkably, we did not observe a strong interaction by gel filtration between MATβV2 with MATα1 (Fig. S4C), indicating a role of the N-terminus of MATβ in providing the stability of the MATα1β complex. To confirm this hypothesis, we generated a truncated version of MATβV1 lacking the first 16 amino acids (MATβV1Δ16). This mutant produces a much smaller amount of complex with MATα1, supporting the hypothesis that the MATβ N-terminus is indeed important for the formation of stable MATα1β complexes (Fig. S4D).

### 2.4. Active site and enzymatic activity   

**Figure 6:**
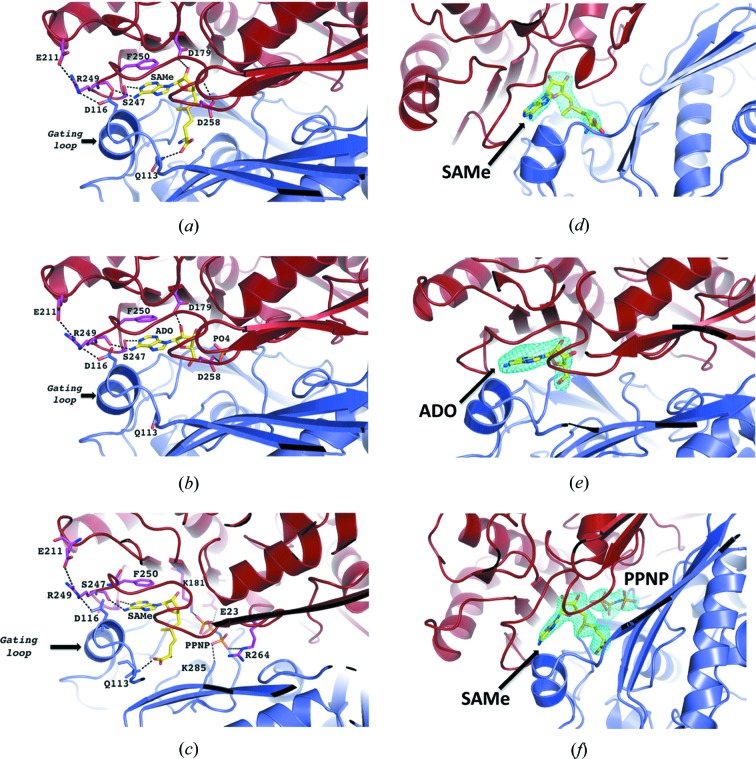
Active site. MATα2 monomer with visible gating loop is coloured in slate whereas MATα2 monomer with disordered loops is coloured in red. Important hydrogen bonds are shown as dotted lines. (*a*) Stick representation of the bound product, SAMe, at the active site. (*b*) Crystal co-crystallized and soaked with only ATP shows adenosine molecule (ADO) and PO_4_ at the active site, as a result of the ATPases and tripolyphosphatase activity of MATα2. (*c*) Crystal co-crystallized and soaked with AMPPNP and methionine shows SAMe and PPNP. (*d*) Omit (*F*_o_ − *F*_c_) electron-density map and stick representation of bound molecule, the map is contoured at the 2.5σ level around the SAMe molecule, (*e*) around the adenosine (ADO) molecule and (*f*) around SAMe and PPNP.

After incubation of the MAT(α2)_4_(βV2)_2_ complex with its product SAMe, its substrate MET, ATP or AMPPNP (non-hydrolysing ATP analogue), the presence of SAMe, adenosine (ADO) or PPNP [(β-γ-imido)triphosphate] was clearly observed in different crystals at the active site (Figs. 6 ▶ *a*, 6 ▶ *b* and 6 ▶ *c*) as supported by the corresponding omit maps (Figs. 6 ▶ *d*, 6 ▶ *e* and 6 ▶ *f*). In all of the structures the adenine group makes a π–π stacking interaction with F250 of MATα2, supporting the hypothesis that the substrate (ATP) and product (SAMe) can occupy the active site in similar orientations. The structures show that two of the four active sites are occupied by SAMe or adenosine whereas the other two are empty, thus providing details of structural differences that accompany SAMe formation by comparison of empty and occupied sites.

**Figure 7:**
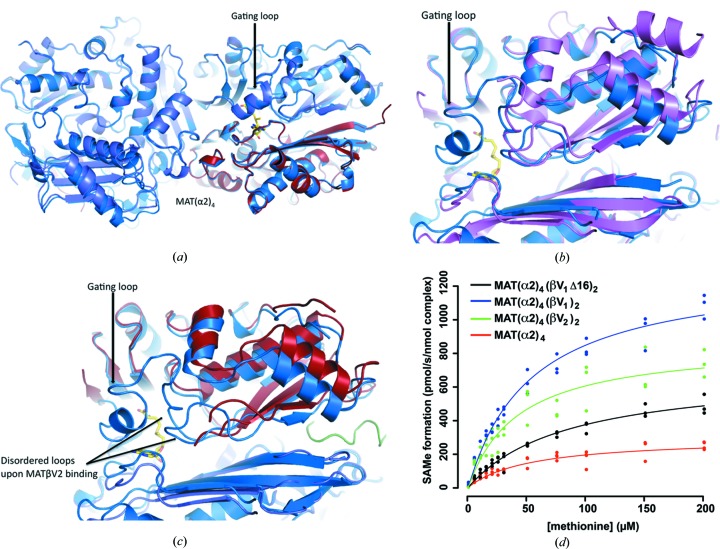
Comparison of MAT(α2)_4_(βV2)_2_ complex with MAT(α2)_4_. (*a*) Superposition of SAMe-bound MAT(α2)_4_ (PDB entry 2p02 in blue) and SAMe-bound MAT(α2)_4_ (slate, red) from the complex MAT(α2)_4_(βV2)_2_; in the closed state both structures are similar. (*b*) Superposition of apo-MAT(α2)_2_ from *Burkholderia pseudomallei* (PDB entry 3iml, Baugh *et al.*, 2013 ▶, in pink) with the SAMe-bound MAT(α2)_2_ (PDB entry 2p02 in blue); in the absence of SAMe only the gating loop is disordered. (*c*) Superposition of SAMe-bound MAT(α2)_2_ (PDB entry 2p02 in blue) with MAT(α2)_2_ after MATβV2 binding; the open state in this case shows two additional flexible loops, near the inserted MATβV2 C-terminus. (*d*) ATP was added to MAT(α2)_4_, MAT(α2)_4_(βV1Δ16)_2_, MAT(α2)_4_(βV2)_2_ and MAT(α2)_4_(βV1)_2_ pre-incubated with 5–200 µ*M* of methionine then SAMe formation was quantified using UPLC–MS/MS. It shows that the *V*_max_ of MATα2β complexes are higher depending on the variant of MATβ.

A comparison of the active site in two different states reveals that loops flanking the empty catalytic pockets are disordered (Fig. 2 ▶ *a*), in particular the ‘gating loop’ (residues 113–131) that has been proposed to act as a dynamic lid controlling access to the active site (Komoto *et al.*, 2004 ▶). The flexibility of this loop induces two different conformations of the catalytic subunit. The loop is disordered in the open conformation causing the entrance to the active site to be opened. The entrance to the active site is blocked in the closed conformation and the gating loop becomes well ordered (Fig. 7 ▶ *a*). In the case of the catalytic subunit MATα1, *S*-nitrosylation of residue C121 in the ‘gating loop’ promotes the inactivation of the enzyme (Pérez-Mato *et al.*, 1999 ▶). A comparison of the SAMe-bound MAT(α2)_4_(βV2)_2_ complex with SAMe-bound MAT(α2)_4_ (PDB entry 2p02; Shafqat *et al.*, 2013 ▶) shows that in the absence of MAT(βV2)_2_ the four active sites of MAT(α2)_4_ are in a closed conformation. The complex formation with MAT(βV2)_2_ causes an asymmetry in MAT(α2)_4_, in which two sites are found in an open state while the other two are in a closed conformation (Fig. 1 ▶ *a*). In the absence of SAMe, the apo-structures exhibit no density for the gating loop, indicating its flexible nature (Fig. 7 ▶ *b*). However in MAT(α2)_4_(βV2)_2_ the open active sites show two additional flexible loops, near the inserted MATβV2 C-terminus (Fig. 7 ▶ *c*). In addition, the N-terminal loop (residues 1–13) of both MATβV2 in the complex, which is orientated to the same side of the open active sites, is disordered. This observation raises the question of whether the N-terminus of MATβ could regulate the gating loop of MATα2. If this was the case, the differences between the N-terminus of MATα2βV1 and MATα2βV2 should affect their enzymatic activities. Thus, we compared the activity of the MAT(α2)_4_(βV1)_2_, MAT(α2)_4_(βV2)_2_ and MAT(α2)_4_(βV1Δ16)_2_ complexes. Notably, the presence of either MATβV1 or MATβV2 increased the *V*_max_ of MAT(α2)_4_ without altering the *K*_m_ for methionine. Additionally, the *V*_max_ of MATα2 was 34% higher in the presence of MATβV1 than with MATβV2, thus emphasising that differences at the N-terminus affect the activity of MAT(α2)_4_. Furthermore, the MAT(α2)_4_(βV1Δ16)_2_ complex, in which MATβ has the shortest N-terminus, also has the lowest *V*_max_ which is still higher than the catalytic subunit alone (Fig. 7 ▶ *d* and Table 3 ▶). Therefore, the increasing N-terminal length correlates with an increase in *V*_max_ of the complex activity, confirming the role of the N-terminal in the regulation of the activity.

A more extensive mutational analysis shows by gel filtration that the minimum motif required for the formation of the MAT(α2)_4_(βV2)_2_ complex comprises three residues at the end of the C-terminal of MATβV2 (Val_321_Phe_322_His_323_). This motif interacts with the loop that recognizes the tripolyphosphate of the ATP at the active site, suggesting a possible allosteric mechanism that could be responsible for the observed increase of the *V*_max_ of MAT(α2)_4_(βV1Δ16)_2_ in comparison with the catalytic subunit alone (Fig. 4 ▶ *c*).

In summary, we propose that the N-terminal loop of MATβ acts in a concerted manner with the C-terminus motif, helping the release of the product by making the active site solvent-accessible for the next substrate to be processed. MATα2β activity and SAMe levels appear to be tightly linked with cell proliferation, *e.g.* upregulation of MATα2 and/or MATβ provides a growth advantage in hepatoma and colon cancer cells (Attia *et al.*, 2008 ▶; Yang *et al.*, 2008 ▶; Lu & Mato, 2012 ▶; Xia *et al.*, 2010 ▶; Chen *et al.*, 2007 ▶). Similarly, T-leukaemic cells exhibit higher MATα2β activity but, remarkably, when MATα2 expression is inhibited SAMe levels decrease and there is more apoptosis (Attia *et al.*, 2008 ▶; Jani *et al.*, 2009 ▶). In this regard, regulating SAMe production might be an option for potential anticancer therapies. The structure of the MAT(α2)_4_(βV2)_2_ complex presented here has direct implications for a broad range of SAMe-based biochemistry (Landgraf & Booker, 2013 ▶; Kim *et al.*, 2013 ▶). Our results show that the complex MAT(α1)_4_(βV1)_2_ is stable *in vitro*, raising the possibility that this complex may exist during the transition of the expression between different isoforms. It also provides a gateway for structure-based drug design with the aim of searching lead compounds that regulate the levels of SAMe without interfering with the catalytic reaction for its synthesis.

## 3. Materials and methods   

### 3.1. Protein expression and purification   

MATα1, MATα2 constructs were kindly provided by SGC Oxford and MATβV1, MATβV2 constructs were from the laboratory of SCL. MATβV1 and MATβV1Δ16 isoforms were cloned in the HIS-parallel vector *via Nco*I and *Xho*I sites, and MATβV2 was cloned in the pET-28a(+) vector *via* *Nde*l and *Xho*l sites. The expression of MATβV1 was carried out in *Escherichia coli* BL21(HC41) and the expression of the other three proteins in *E. coli* BL21(DE3) strain. Cells were grown in LB medium at 37°C to an *A*_600_ = 0.6–0.8 at which protein expression was induced by the addition of 1 m*M* isopropyl β-d-1-thiogalactopyranoside (IPTG) (GoldBio), at 20°C overnight.

Cell pellets were lysed at 4°C using high-pressure homogenization at 27 Kpsi (1 Kpsi = 69 × 10^3^ MPa) (Constant System Ltd, UK) in lysis buffer (500 m*M* NaCl, 5% glycerol, 5 m*M* imidazole, 10 m*M* BME (β-mercaptoethanol) and the cell homogenate was clarified by centrifugation at 20 000 rev min^−1^ for 40 min. The clarified supernatant was loaded onto nickel resin equilibrated with lysis buffer. The column was washed with lysis buffer and then with wash buffer (500 m*M* NaCl, 5% glycerol, 30 m*M* imidazole, 10 m*M* BME). Proteins were eluted with elution buffer (500 m*M* NaCl, 250 m*M* imidazole, 10 m*M* BME) and the His tag was then cleaved from MATα2, MATα1 and MATβV1 by incubation overnight with *Tobacco etch virus* (TEV) protease and with thrombin in the case of MATβV2. MATα2, MATα1 and MATβV2 were then loaded onto an ion-exchange chromatography column (HiTrap Q HP column, GE Healthcare), and MATβV1 on a HiTrap S HP, that were pre-equilibrated with buffer *A* (50 m*M* NaCl, 5 m*M* BME); purification was then performed using an isocratic gradient from 0.05 to 1 *M* NaCl. Selected fractions of MATα2 and MATα1 were then concentrated and loaded onto a HiLoad 16/60 Superdex 200 gel-filtration column (GE Healthcare) and fractions of MATβV2 and MATβV1 onto a HiLoad 16/60 Superdex 75. Finally, fractions containing pure protein were pooled and stored at −80°C. The buffer used in each purification step was 25 m*M* HEPES pH 7.5.

### 3.2. Complex formation, crystallization and data collection   

In order to assemble the complexes, MATα and MATβ were incubated together for 1 h at 4°C in 50 m*M* HEPES buffer pH 7.5 containing 10 m*M* MgCl_2_, 50 m*M* KCl, 300–500 µ*M* NADP. The complex was then loaded onto a Superdex 200 10/300 column and eluted with buffer consisting of 200 m*M* NaCl, 25 m*M* HEPES pH 7.5, 1 m*M* MgCl_2_, 5 m*M* KCl, 1 m*M* TCEP [tris(2-carboxyethyl)phosphine]. Crystals appeared at 25°C within 1–2 d in drops containing 2 µl MAT(α2)_4_(βV2)_2_ complex at 5.8 mg ml^−1^ mixed with 1 µl precipitant solution of 100 m*M* MES/imidazole buffer pH 6.5, 10% ethylene glycol, 20% PEG 8K. Before crystallization the MAT(α2)_4_(βV2)_2_ complex was incubated with its product SAMe (1 m*M*), its substrate ATP (1 m*M*) or AMPPNP (250 µ*M*) and MET (1 m*M*). These compounds were added to the precipitant and cryosolution. Different data sets were collected at the PROXIMA1, XALOC and I04 beamlines at SOLEIL (St Aubin, France), ALBA (Barcelona, Spain) and Diamond (Oxford, England) synchrotron centres, respectively. Data reduction was carried out with the *HKL*-2000 (Otwinowski & Minor, 1997 ▶) and *XDS* programs (Kabsch, 2010 ▶). The phases were calculated with *Phaser* (McCoy *et al.*, 2007 ▶) using MATα2 (PDB entry 2p02) and MATβ (PDB entry 2ydy, Shafqat *et al.*, 2013 ▶) as search models for molecular replacement. Model building and refinement were performed using *Coot* (Emsley & Cowtan, 2004 ▶), *PHENIX* (Adams *et al.*, 2010 ▶) and *REFMAC* (Murshudov *et al.*, 2011 ▶). Data-collection and refinement statistics are summarized in Table 4 ▶.

SAXS data were collected on the SWING beamline at the SOLEIL synchrotron, using the HPLC-integrated SAXS set-up with a two-dimensional AVIEX CCD detector over an angular range *q*_min_ = 0.01 Å^−1^ to *q*_max_ = 0.5 Å^−1^. 80 µl MAT(α2)_4_(βV2)_2_ complex at 5 mg ml^−1^ was loaded onto a pre-equilibrated Shodex KW-402.5-4F 150 kDa SEC (size-exclusion chromatography) column, 250 frames of SAXS data were taken over the course of protein elution. Data averaging and reduction were carried out with the *Foxtrot* suite, developed at SOLEIL for the SWING beamline. Further analyses were performed with the *ATSAS* suite (Petoukhov *et al.*, 2012 ▶). In order to generate an *ab initio* model ten runs of *DAMMIN* (Svergun, 1999 ▶) were performed, and after averaging and filtering a model with 732 beads was produced.

### 3.3. Isothermal titration calorimetry (ITC)   

In order to address the association constant (*K*_a_) of MATαβ complexes, MATα2, MATα1, MATβV1, MATβV2 and the mutant variants were buffer-exchanged by gel filtration on a Superdex 200 10/300 GL column equilibrated with 200 m*M* NaCl, 20 m*M* HEPES pH 7.5 buffer before ITC analysis. Subsequently, MATβ isoforms and mutants were injected into MATα2 or MATα1 solution in aliquots of 10 or 20 µl, respectively (Table 1 ▶). To verify the interaction of NADP with different MATβ isoforms, each protein was buffer-exchanged by gel filtration on a Superdex 200 10/300 GL column equilibrated in 5 m*M* MgCl_2_, 5 m*M* KCl, 20 m*M* HEPES pH 7.5, 200 m*M* NaCl buffer before ITC analysis. NADP was also diluted in the same buffer. NADP was injected into MATβV2 in aliquots of 15 µl and into MATβV1 in aliquots of 10 µl (Table 2 ▶). All ITC measurements were carried out at 25°C on a VP-ITC Microcalorimeter (MicroCal/GE Healthcare). The ITC data were processed using *Origin* software (OriginLab Corp., USA).

### 3.4. Activity assays   

MAT activity was addressed by measuring production of SAMe using a fixed concentration of ATP (1 m*M*) and different concentrations of methionine (5–200 µ*M*). The concentration of MATα2 and MATαβ complexes was optimized to evaluate the activity at the linear region of the Michaelis curve. The final concentrations of proteins in the reaction were 50, 25 and 12.5 n*M* for MATα2, MAT(α2)_4_(βV1)_2_ and MAT(α2)_4_(βV2)_2_, respectively.

The reaction was carried out in a final volume of 200 µl. The mixture contained 40 µl of the enzyme [250 n*M* for MATα2, 125 n*M* for MAT(α2)_4_(βV1)_2_ and 62.5 n*M* for MAT(α2)_4_(βV2)_2_], 40 µl of methionine (25–1000 µ*M*) and 40 µl of ATP (5 m*M*) in 80 µl of reaction buffer (50 m*M* HEPES pH 7.5, 25 m*M* MgCl_2_, 25 m*M* KCl). The blank control was prepared in the absence of ATP. All stock solutions for methionine, ATP and each enzyme were prepared in the dilution buffer [10 m*M* HEPES pH 7.5, 500 m*M* NaCl, 5%(*v*/*v*) glycerol, 0.5 m*M* TCEP].

For each reaction the protein, methionine and buffer were pre-incubated for 15 min prior to the addition of ATP. The reaction mixtures were thermostated and agitated (37°C, 1400 rev min^−1^). After 10 min the reactions were terminated by the addition of 800 µl of 75% acetonitrile and 1.2% formic acid. To ensure the reaction stopped after the addition of acetonitrile/formic acid solution all samples were shaken (4°C, 1400 rev min^−1^) before centrifugation (14 000 rev min^−1^) and transferred to 96-well plates for UPLC–MS analysis.

Samples were injected in a randomized order for the detection and quantification of SAMe and methionine. Briefly, upon injection, polar metabolites bind to the UPLC column and are then eluted on a polarity gradient. Each fraction was subjected to mass-spectroscopy analysis to determine the production of SAMe and the remaining methionine levels. A ten-point calibration curve with exponentially spaced concentrations of methionine was used for quantization of each sample (van Liempd *et al.*, 2013 ▶). The rate of SAMe formation was calculated (pmol s^−1^ per nmol of complex or pmol s^−1^ per nmol of MAT2α tetramer) for each substrate concentration. *R* (R Core Team, 2013 ▶) software was used to fit the enzyme kinetic data with the Michaelis–Menten equation for calculation of *V*_max_ and *K*_m_ values. Each reaction was performed in triplicate.

## Supplementary Material

PDB reference: SAMe-bound, 4ktt

PDB reference: ADO-bound, 4ktv

PDB reference: imido-triphosphate-bound, 4ndn

Supporting information.. DOI: 10.1107/S2052252514012585/lz5002sup1.pdf

## Figures and Tables

**Table 1:** Thermodynamic parameters of MATαβ complex formation

	MATβV2 → MATα2	MATβV1 → MATα2	MATβV1 → MATα1
[]	36.5 µ*M* → 10 µ*M*	51.2 µ*M* → 10 µ*M*	236.8 µ*M* → 45.1 µ*M*
*K*_a_ (*M* ^−1^)	(1.14 ± 0.17) × 10^8^	(2.90 ± 0.82) × 10^7^	(3.38 ± 0.24) × 10^5^
*K*_d_ (*M*)	(8.77 ± 1.34) × 10^−9^	(3.44 ± 0.98) × 10^−8^	(2.96 ± 0.21) × 10^−6^
Δ*H*	−2.28 × 10^4^ ± 188.4	−2.37 × 10^4^ ± 367.6	−9301± 140.9
*N*	0.44 ± 0.001	0.44 ± 0.004	0.325 ± 0.004

**Table 2:** Thermodynamic parameters of NADP binding to MATβ isoforms

	NADP → MATβV2	NADP → MATβV1	NADP → MAT(α2)_4_(βV2)_2_
[]	500 µ*M* → 30 µ*M*	500 µ*M* → 29.8 µ*M*	155 µ*M*→ 10.22 µ*M*
*K*_a_ (*M* ^−1^)	(1.12 ± 0.13) × 10^5^	(1.17 ± 0.22) × 10^5^	
*K*_d_ (*M*)	(8.93 ± 1.07) × 10^−6^	(8.55 ± 1.6) × 10^−6^	
Δ*H*	2784 ±117.4	1866 ± 127.8	
*N*	0.85 ± 0.026	1.23 ± 0.060	

**Table 3:** Kinetic properties of MATα2β complexes

	MAT(α2)_4_	MAT(α2)_4_(βV1Δ16)_2_	MAT(α2)_4_(βV2)_2_	MAT(α2)_4_(βV1)_2_
*k*_cat_	295.08 ± 23.7	688.79 ± 38.39	859.21 ± 57.14	1298.16 ± 47.78
*k*_m_	53.84 ± 10.5	84.79 ± 9.93	42.45 ± 7.38	53.48 ± 4.79

**Table 4:** Data-collection and refinement statistics Values in parentheses are for the highest resolution shell.

	SAMe-bound	ADO-bound	PPNP-bound
Data collection			
Wavelength (Å)	0.98	0.978	0.979
Detector	PILATUS 6M	PILATUS 6M	PILATUS 6M
Space group	*P*2_1_2_1_2_1_	*P*2_1_2_1_2_1_	*P*2_1_2_1_2_1_
Unit-cell dimensions (*a*, *b*, *c*) (Å)	72.44, 115.72, 298.45	72.09, 116.57, 299.48	72.14, 122.18, 298.42
Resolution (Å)	50–2.6 (2.69–2.6)	108.63–3.3 (3.48–3.3)	50–2.35 (2.43–2.35)
*R*_merge_ (%)	14.4 (59.4)	13.9 (49.4)	10.1 (70.4)
*I*/σ*I*	10.12 (1.75)	6.8 (2.7)	17.3 (1.6)
Completeness (%)	98.7 (90.1)	99.9 (99.9)	98.0 (83.2)
Redundancy	5.6 (4.5)	3.3 (3.5)	7.5 (5.2)
Refinement			
Resolution (Å)	2.6	3.3	2.35
No. of reflections	76590	38907	108283
*R*_work_/*R*_free_	21.8/27.9	17.6/26.0	21.1/25.1
No. of atoms			
Protein	15061	16370	16463
Ligand/ion	54/1	38/1	54/4
Water	108	37†	498
*B* factors (Å^2^)			
Protein	76.64	71.19	58.4
Ligand/ions	SAMe/Mg 68.16/60.62	ADO/Mg 97.95/49.14	SAMe/PNPP/Mg 47.73/63.48/39.57
Waters	56.13	30.67†	48.39
Ramachandran statistics			
Residues in preferred regions	1920 (94%)	1863 (90%)	2001 (96%)
Residues in allowed regions	108 (5%)	185 (9%)	76 (4%)
Outliers	10 (0.5%)	20 (1%)	8 (0.38%)
R.m.s. deviations			
Bond lengths (Å)	0.006	0.008	0.005
Bond angles (°)	1.091	1.265	1.026

† The waters in this structure (3.3 Å) were assigned using the high-resolution (2.35 Å) model as reference. Compared to other higher resolution structures very few were assigned (37 compared to 498 in 2.35 Å structure). Many of these waters are located/trapped at the interface of MATα2 dimers accounting for the much lower *B* factors.
