# Supplementary material for: Structure and function study of the complex that synthesizes S-adenosylmethionine
Source: IUCrJ. 2014 Jun 12;1(Pt 4):240–9. doi: 10.1107/S2052252514012585 (PMC4107924; doi:10.1107/S2052252514012585)
Supplement: Supplementary file 1 [file m-01-00240-sup1.pdf]

IUCrJ

Volume 1 (2014)

Supporting information for article:

Structure and function study of the complex that synthesizes *S*-adenosylmethionine

Ben Murray, Svetlana V. Antonyuk, Alberto Marina, Sebastian M. Van Liempd, Shelly C. Lu, Jose M. Mato, S. Samar Hasnain and Adriana L. Rojas

Supporting information

S1. Material and methods

S1.1. Mutagenesis

Site-Directed mutagenesis was carried out in both the His-Parallel-MATβV1 and PET28-MATβV2 vectors using the Quikchange method (Stratagene). Primers used are described in table S5. Sequencing of all the plasmids was performed by the external service of CNIO (Centro Nacional de Investigaciones Oncologicas, Madrid).

S1.2. Circular Dichroism (CD)

CD data was recorded on a J-815 CD spectrometer (*Jasco, UK*) equipped with thermoelectric temperature control. Samples of MATβV1 and MATβV2 and the mutant variants were diluted to 1-5 μM in 0.1 M Phosphate buffer pH 7.0 and placed in a 2-mm cell. All CD wavelength scans were performed at a constant temperature of 25 ºC between 200 and 280 nm with a scanning speed of 50 nm ⁄ min and a data pitch of 1 nm. Data analysis was done using Origin and JASCO Spectra Manager integrated software. Raw data was converted to Ellipticity*10^{-3} (deg cm^2 $dmol^{-1}$) using Microsoft excel.

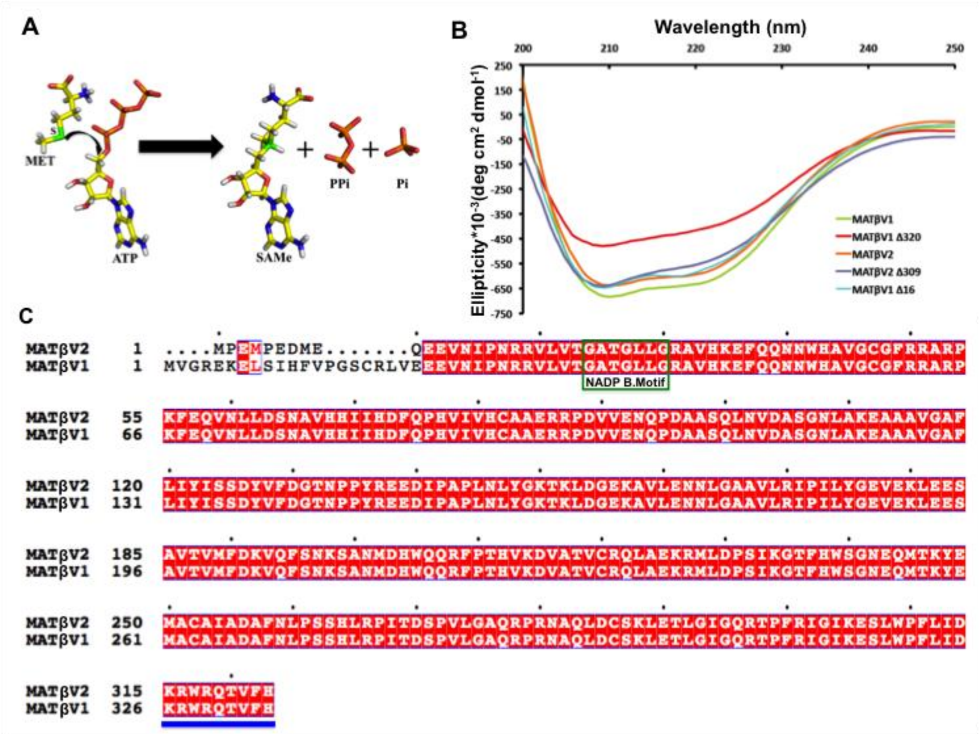

Figure S1 **Schematic representation of SAMe synthesis, Circular Dichroism (CD) spectrums of MATβ proteins and sequence alignment of MATβ isoforms.** (A) The formation of SAMe occurs as an SN_2 reaction with the nucleophilic attack of the methionine sulphur to the ATP C5′ atom. Then, the intermediate tripolyphosphate (PPPi) is hydrolyzed to pyrophosphate (PPi) and orthophosphate (Pi). (B) CD spectrums of MATβV1 (green), MATβV2 (orange), MATβV1Δ320 (red), MATβV2Δ309 (violet) and MATβV1Δ16 (blue). Comparison between the CD spectrums of the wild type and the mutants shows that the secondary structure is conserved after the deletion of the C-terminus (MATβV1Δ320 and MATβV2Δ309) or the N-terminus (MATβV1Δ16). (C) Sequence alignment of MATβV1 and MATβV2, residues that interact with MATα dimer are highlighted in blue and the NADP binding motif is also shown.

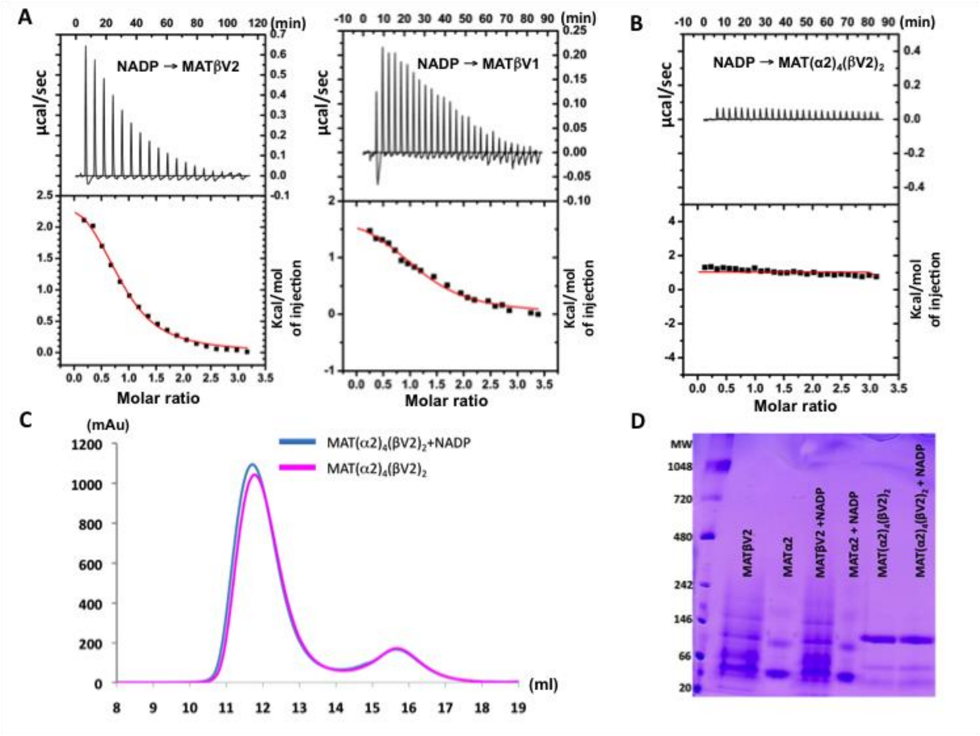

Figure S2 **Interaction of MATα2β complexes with NADP. (A,B)** Isothermal titration calorimetry of MATβV2, MATβV1 and MAT(α2)$_4$(βV2)$_2$ complex with NADP. The top graphs represent the differential heat released during the titration of NADP with the different MAT subunits, the bottom graphs represent the fitted binding isotherms. **(C)** Gel filtration of MAT(α2)$_4$(βV2)$_2$ complex with (blue) and without (magenta) NADP incubation and **(D)** Native-PAGE of MAT subunits and MAT(α2)$_4$(βV2)$_2$ complex with and without NADP.

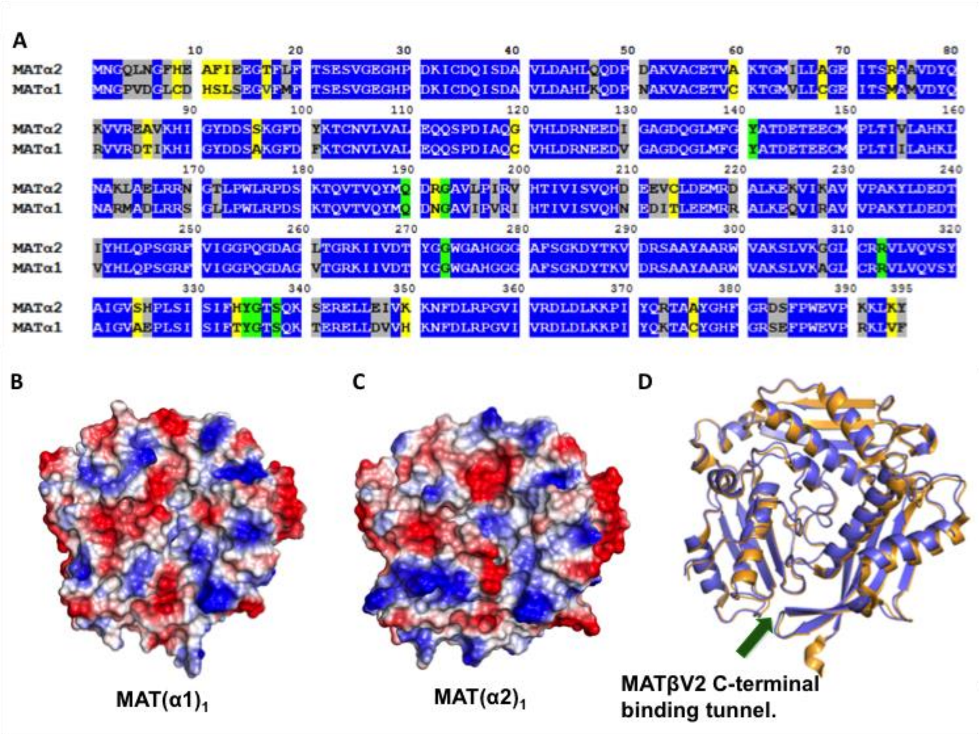

Figure S3 **Comparison between MATα1 and MATα2. (A)** Sequence alignment of the two catalytic MATα subunits, in blue are residues that are equal, in green the residues of MATα2 that interact with MATβV2, in grey are residues that are similar and in yellow the residues that are different. **(B)** Electrostatic surface representation of MATα1 and **(C)** MATα2 shows different charge distribution at the surface of both proteins. Negative charge is shown in red colour, positive in blue. The surface potential has been calculated with Pymol/APBS. **(D)** Superposition of the MATα1 monomers (PDB:2OBV in orange) and MATα2 from the MAT$(\alpha 2)_4(\beta V2)_2$ complex (slate), the green arrow represents the region where MATβ C-terminal interacts.

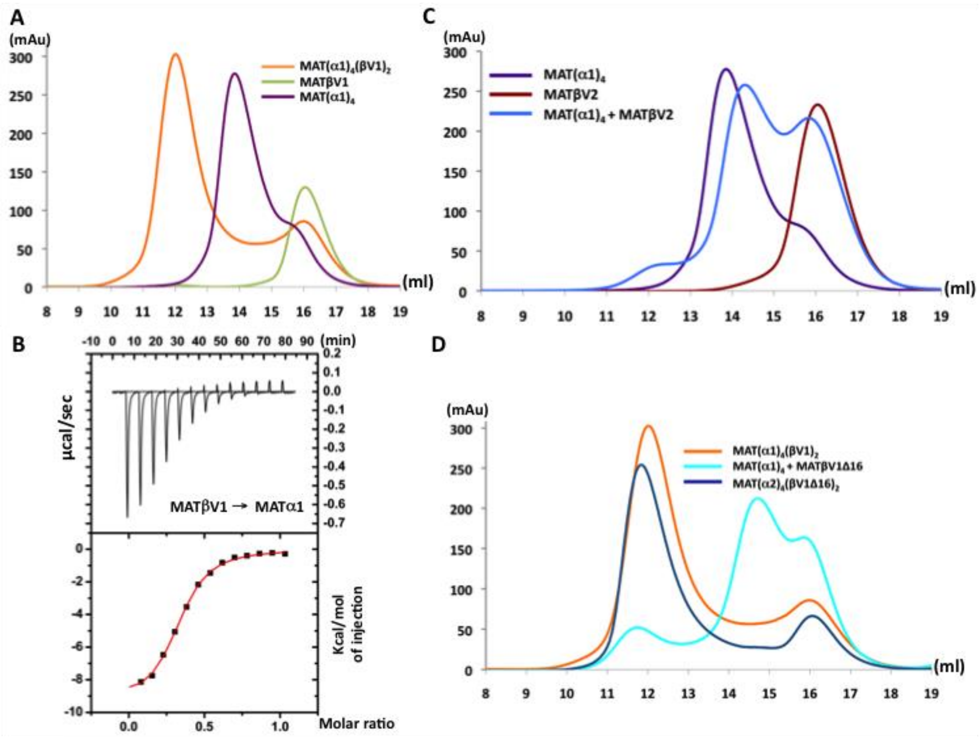

Figure S4 Gel filtration (GF) and ITC of MATαβ complexes. **(A)** MATβV1 (green) and MAT(α1)$_4$ (purple) were loaded in the GF column as a control in the same buffer of the MAT(α1)$_4$(βV1)$_2$ complex (orange). **(B)** ITC of MATα1 with MATβV1, the top graph represents the differential heat released during the titration MATβV1 with MATα1. The bottom graph represents the fitted binding isotherms. **(C)** MATβV2 (dark red) and MAT(α1)$_4$ (purple) were loaded in the GF column as a control in the same buffer of the mix of both proteins (blue). Presence of a shoulder on the blue curve at ~12 ml indicates the presence of small amount of the MAT(α1)$_4$(βV2)$_2$ complex. **(D)** The gel filtration profile shows that the formation of a MATαβ complex when MATα2 is mixed with MATβV1Δ16 (dark blue) is significantly larger when compared to when MAT(α1)$_4$ is used (cyan). MAT(α1)$_4$(βV1)$_2$ complex was loaded as a control (orange).

Table S1 **Data collection and refinement statistics**. *Highest resolution shell is shown in parenthesis

	SAMe-bound	ADO-bound	PPNP-bound
**Data collection**			
Wavelength	0.98	0.978	0.979
Detector	Pilatus 6M	Pilatus 6M	Pilatus 6M
Space group	$P2_12_12_1$	$P2_12_12_1$	$P2_12_12_1$
Cell dimensions			
a, *b*, *c* (Å)	72.44, 115.72, 298.45	72.09, 116.57, 299.48	72.14, 122.18, 298.42
Resolution (Å)	50-2.6(2.69-2.6) *	108.63-3.3 (3.48-3.3) *	50-2.35 (2.43-2.35) *
R_{merge} (%)	14.4 (59.4)	13.9 (49.4)	10.1 (70.4)
$I/\sigma I$	10.12 (1.75)	6.8 (2.7)	17.3 (1.6)
Completeness (%)	98.7 (90.1)	99.9 (99.9)	98.0 (83.2)
Redundancy	5.6 (4.5)	3.3 (3.5)	7.5 (5.2)
**Refinement**			
Resolution (Å)	2.6	3.3	2.35
No. reflections	76590	38907	108283
R_{work}/R_{free}	21.8/27.9	17.6/26.0	21.1/25.1
No. atoms			
Protein	15061	16370	16463
Ligand/ion	54 / 1	38 / 1	54 / 4
Water	108	37	498
**B-factors (Å^2)**			
Protein	76.64	71.19	58.4
Ligand/ions	SAMe / Mg	ADO / Mg	SAMe /PNPP /Mg
	68.16 / 60.62	97.95/49.14	47.73 / 63.48 /39.57
Waters	56.13	30.67	48.39
**Ramachandran Statistics**			
Residues in Preferred Regions	1920 (94%)	1863 (90%)	2001 (96%)
Residues in Allowed regions	108 (5%)	185 (9%)	76 (4%)
Outliers	10(0.5%)	20 (1%)	8 (0.38%)
R.m.s deviations			
Bond lengths (Å)	0.006	0.008	0.005
Bond angles (º)	1.091	1.265	1.026

Table S2 Primers used in this study.

**MATβV1W320Stop and MATβV2W309Stop**
Forward:
5' GGAATCAAAGAATCACTTTAGCCTTTCCTCATTGACAAG 3'
Reverse:
5' CTTGTCAATGAGGAAAGGCTAAAGTGATTCTTTGATTCC 3'
**MatβV1Δ16**
Forward
5´ TTTTTCCATGGGACTCTCTATACACTTTGTTCCCGGG ´3
Reverse
5´ TTTTTCTCGAGCTAATGAAAGACCGTTTG ´3
